# Combined Impact of Lifestyle-Related Factors on Total and Cause-Specific Mortality among Chinese Women: Prospective Cohort Study

**DOI:** 10.1371/journal.pmed.1000339

**Published:** 2010-09-14

**Authors:** Sarah J. Nechuta, Xiao-Ou Shu, Hong-Lan Li, Gong Yang, Yong-Bing Xiang, Hui Cai, Wong-Ho Chow, Butian Ji, Xianglan Zhang, Wanqing Wen, Yu-Tang Gao, Wei Zheng

**Affiliations:** 1 Division of Epidemiology, Department of Medicine, Vanderbilt University School of Medicine, Nashville, Tennessee, United States of America; 2 Department of Epidemiology, Shanghai Cancer Institute, Shanghai, China; 3 Division of Cancer Epidemiology and Genetics, National Cancer Institute, Rockville, Maryland, United States of America; University of Cambridge, United Kingdom

## Abstract

**Background:**

Although cigarette smoking, excessive alcohol drinking, obesity, and several other well-studied unhealthy lifestyle-related factors each have been linked to the risk of multiple chronic diseases and premature death, little is known about the combined impact on mortality outcomes, in particular among Chinese and other non-Western populations. The objective of this study was to quantify the overall impact of lifestyle-related factors beyond that of active cigarette smoking and alcohol consumption on all-cause and cause-specific mortality in Chinese women.

**Methods and Findings:**

We used data from the Shanghai Women's Health Study, an ongoing population-based prospective cohort study in China. Participants included 71,243 women aged 40 to 70 years enrolled during 1996–2000 who never smoked or drank alcohol regularly. A healthy lifestyle score was created on the basis of five lifestyle-related factors shown to be independently associated with mortality outcomes (normal weight, lower waist-hip ratio, daily exercise, never exposed to spouse's smoking, higher daily fruit and vegetable intake). The score ranged from zero (least healthy) to five (most healthy) points. During an average follow-up of 9 years, 2,860 deaths occurred, including 775 from cardiovascular disease (CVD) and 1,351 from cancer. Adjusted hazard ratios for mortality decreased progressively with an increasing number of healthy lifestyle factors. Compared to women with a score of zero, hazard ratios (95% confidence intervals) for women with four to five factors were 0.57 (0.44–0.74) for total mortality, 0.29 (0.16–0.54) for CVD mortality, and 0.76 (0.54–1.06) for cancer mortality. The inverse association between the healthy lifestyle score and mortality was seen consistently regardless of chronic disease status at baseline. The population attributable risks for not having 4–5 healthy lifestyle factors were 33% for total deaths, 59% for CVD deaths, and 19% for cancer deaths.

**Conclusions:**

In this first study, to our knowledge, to quantify the combined impact of lifestyle-related factors on mortality outcomes in Chinese women, a healthier lifestyle pattern—including being of normal weight, lower central adiposity, participation in physical activity, nonexposure to spousal smoking, and higher fruit and vegetable intake—was associated with reductions in total and cause-specific mortality among lifetime nonsmoking and nondrinking women, supporting the importance of overall lifestyle modification in disease prevention.

*Please see later in the article for the Editors' Summary*

## Abstract 2

Findings from the Shanghai Women's Health Study confirm those derived from other, principally Western, cohorts regarding the combined impact of lifestyle-related factors on mortality.

## Editors' Summary

**Background:**

It is well established that lifestyle-related factors, such as limited physical activity, unhealthy diets, excessive alcohol consumption, and exposure to tobacco smoke are linked to an increased risk of many chronic diseases and premature death. However, few studies have investigated the combined impact of lifestyle-related factors and mortality outcomes, and most of such studies of combinations of established lifestyle factors and mortality have been conducted in the US and Western Europe. In addition, little is currently known about the combined impact on mortality of lifestyle factors beyond that of active smoking and alcohol consumption.

**Why Was This Study Done?:**

Lifestyles in regions of the world can vary considerably. For example, many women in Asia do not actively smoke or regularly drink alcohol, which are important facts to note when considering practical disease prevention measures for these women. Therefore, it is important to study the combination of lifestyle factors appropriate to this population.

**What Did the Researchers Do and Find?:**

The researchers used the Shanghai Women's Health Study, an ongoing prospective cohort study of almost 75,000 Chinese women aged 40–70 years, as the basis for their analysis. The Shanghai Women's Health Study has comprehensive baseline data on anthropometric measurements, lifestyle habits (including the responses to validated food frequency and physical activity questionnaires), medical history, occupational history, and select information from each participant's spouse, such as smoking history and alcohol consumption. This information was used by the researchers to create a healthy lifestyle score on the basis of five lifestyle-related factors shown to be independently associated with mortality outcomes in this population: normal weight, lower waist-hip ratio, daily exercise, never being exposed to spouse's smoking, and higher daily fruit and vegetable intake. The score ranged from zero (least healthy) to five (most healthy) points. The researchers found that higher healthy lifestyle scores were significantly associated with decreasing mortality and that this association persisted for all women regardless of their baseline comorbidities. So in effect, healthier lifestyle-related factors, including normal weight, lower waist-hip ratio, participation in exercise, never being exposed to spousal smoking, and higher daily fruit and vegetable intake, were significantly and independently associated with lower risk of total, and cause-specific, mortality.

**What Do These Findings Mean?:**

This large prospective cohort study conducted among lifetime nonsmokers and nonalcohol drinkers shows that lifestyle factors, other than active smoking and alcohol consumption, have a major combined impact on total mortality on a scale comparable to the effect of smoking—the leading cause of death in most populations. However, the sample sizes for some cause-specific analyses were relatively small (despite the overall large sample size), and extended follow-up of this cohort will provide the opportunity to further evaluate the impact of these lifestyle-related factors on mortality outcomes in the future.

The findings of this study highlight the importance of overall lifestyle modification in disease prevention, especially as most of the lifestyle-related factors studied here may be improved by individual motivation to change unhealthy behaviors. Further research is needed to design appropriate interventions to increase these healthy lifestyle factors among Asian women.

**Additional Information:**

Please access these Web sites via the online version of this summary at http://dx.doi.org/10.1371/journal.pmed.1000339

- The Vanderbilt Epidemiology Center has more information on the Shanghai Women's Health Study
- The World Health Organization provides information on health in China
- The document Health policy and systems research in Chinacontains information about health policy and health systems research in China
- The Chinese Ministry of Healthalso provides health information

## Introduction

Lifestyle-related factors—such as high adiposity, low or no exercise participation, unhealthy dietary habits, and environmental tobacco smoke—each have been linked to an increased risk of multiple chronic diseases and premature death [1]–[11]. However, few studies have investigated the combined impact of these lifestyle-related factors and mortality outcomes [2],[12]–[15]. Research to quantify the overall impact of lifestyle-related factors on mortality outcomes will provide important information valuable for disease prevention. A recent prospective cohort study of 77,782 participants of the Nurse's Health Study (NHS) found a more than 4-fold increase in risk of all-cause mortality among women aged 34–59 y who reported ever smoking, a body mass index (BMI) ≥25 kg/m^2^, <30 min per day of physical activity, an unhealthy diet score, and heavy or no alcohol drinking, compared to women with none of these risk factors [13]. Another prospective cohort study among 20,244 British men and women aged 45–79 y similarly reported a 4-fold increase in risk of all-cause mortality for participants with no health behaviors compared to participants who had four health behaviors (nonsmoker, plasma vitamin C levels indicative of ≥5 daily servings of fruits and vegetables, moderate alcohol intake, and physically active) [14].

Most studies of combinations of established lifestyle factors and mortality have been conducted in the United States and countries in Western Europe. Data are limited for other populations, including Chinese women, whose lifestyles differ considerably from their European counterparts [16],[17]. Further, active smoking and alcohol consumption, which are two well-studied predictors of mortality [2],[13], have been included in the previous studies. However, many women, and in particular Asian women [17], do not actively smoke or drink regularly, and thus it is important to study practical disease prevention measures for these women. Little is known, however, at present about the combined impact of lifestyle factors beyond that of active smoking and alcohol drinking on mortality.

In the Shanghai Women's Health Study, a population-based cohort study of approximately 75,000 middle-aged and older Chinese women, less than 3% of cohort members reported ever smoking and drinking alcohol regularly, providing a unique opportunity to quantify the overall impact of lifestyle factors other than active smoking and alcohol consumption on total and cause-specific mortality. Well-studied lifestyle-related factors relevant for this population were selected on the basis of prior knowledge of lifestyle factors in relation to mortality and with consideration of practical public health recommendations [1],[3]–[9],[18]–[24]. Specifically, the lifestyle factors selected included: (1) BMI, (2) waist-hip ratio (WHR), (3) exercise participation, (4) environmental tobacco smoke (assessed as exposure to spousal smoking), and (5) fruit and vegetable daily intake.

## Methods

### Study Population

Participants of this analysis are individuals in the Shanghai Women's Health Study (SWHS), an ongoing prospective cohort study of Chinese women. The study methods and rationale have been reported in detail elsewhere [17]. Briefly, participants were recruited from seven urban counties in Shanghai, China. A total of 74,942 women aged 40–70 y were recruited from December 1996 through May 2000 with a participation rate of 92.7%. The baseline survey included an in-person interview, self-administered questionnaire, and anthropometric measurements taken by trained interviewers using standardized protocols. Information was collected on demographics, lifestyle habits (e.g., diet, physical activity, alcohol, smoking), menstrual and reproductive history, medical history, occupational history, and select information from each participant's spouse (e.g., disease history, smoking and alcohol habits). Both the food frequency and physical activity questionnaires have been validated and reported elsewhere [25],[26]. All participants provided written informed consent, and human participant Institutional Review Board (IRB) approval was obtained by the appropriate IRBs in China and the United States.

Follow-up for participants has included in-person interviews every 2–3 y to collect interim health history. Response rates were 99.8%, 98.7%, and 96.7% for the first, second, and third follow-up surveys, respectively. Data on vital status and cancer diagnoses also have been obtained by annual linkage to the population-based Shanghai cancer and vital statistics registries. Outcome data for the present analysis were censored at December 31, 2007.

### Lifestyle-Related Factors

Data from the baseline interview were used to assess the lifestyle factors of interest. We were interested in lifestyle-related factors that are simple to assess and have been well-studied previously in relation to mortality. BMI, a measure of general adiposity, was calculated as measured weight in kilograms divided by measured height in meters squared and categorized using the World Health Organization (WHO) classifications [27]: underweight (<18.5 kg/m^2^), normal weight (18.5–24.99 kg/m^2^), overweight (25–29.99 kg/m^2^), obese (≥30 kg/m^2^). Waist and hip circumference measurements were used to calculate the WHR (waist divided by hip circumference), a measure of central adiposity, and classified into three categories according to tertiles. During the baseline interview, participants were asked about regular exercise in the past 5 y (“regular” was defined as at least once per week, for more than 3 mo continuously). Information was also collected on type, intensity, and duration for up to three activities. We categorized exercise using standard metabolic equivalents (METs) as MET-hours/day (no exercise participation, >0 to 1.99 MET-h/d, and ≥2.0 MET-h/d) [26],[28]. One MET-hour/day is approximately equivalent to about 15 min of participation in moderate-intensity activities [20],[29]. Exposure to environmental tobacco smoke was defined as ever exposed to spousal smoking or never exposed. Grams per day of fruit and vegetable intake were assessed via a food frequency questionnaire for intake over the past 12 mo and categorized into tertiles.

### Statistical Analyses

The primary study outcome was deaths from all causes. Cause of death information was collected from death certificates and coded according to the International Classification of Diseases, 9th Revision (ICD-9). Cause-specific deaths examined included deaths due to cardiovascular disease (CVD) (ICD-9 codes: 390–459) and cancer (ICD-9 codes: 140–208).

Among 74,942 women who completed the baseline assessment, 2,113 reported ever smoking (2.8%) and 1,678 reported ever drinking (2.2%); these women were excluded from the analyses (*n = *3,513). We also excluded women with missing data on the lifestyle factors (anthropometric measures ([*n = *59] and FFQ items for main foods of interest [*n = *11]), with extreme daily energy intake (defined as <500 or ≥3,500 kcal per day) (*n = *108), and who were lost to follow-up shortly after the baseline recruitment (*n = *8). In addition, women who did not have information on exposure to spousal smoking (*n = *7,452) were excluded from analyses of environmental tobacco smoke and the combined effect of lifestyle factors on mortality. We compared select characteristics for women included in the current analyses to women in the entire SWHS cohort (Table S1). With the exception of age, other characteristics were comparable across the three groups: (1) the entire cohort, (2) the cohort after excluding those who met any of the exclusion criteria stated above except exposure to spousal smoking, and (3) the cohort after further excluding women with missing data for spousal smoking. Due to the large sample size, the tests for several characteristics across these three groups were statistically significant. The cohorts included in the current analysis were somewhat younger than the entire cohort, particularly because of the exclusion of those who had missing data on exposure to spousal smoking, which was primarily due to a deceased spouse.

Two healthy lifestyle scores were created on the basis of previous research and public health recommendations [5],[9],[18],[20],[21],[27], as well as consideration of adequate sample sizes for the five lifestyle factors. As shown in Table 1, a point was assigned to each category for the lifestyle factors BMI, WHR, exercise, and daily fruit and vegetable intake (zero [least healthy] to two [most healthy]), while for spousal smoking status a binary variable was used (ever, zero points and never, one point). Healthy lifestyle score 1 was assigned to each woman by summing the points for the five factors, with a possible range of 0–13. Healthy lifestyle score 2 (Table 1) was created by assigning points to simple binary indicators for each of the five factors with one point for having the healthy factor and again summing the points for the five factors to assign a score to each woman (range of 0–5 points). A higher score indicated a healthier lifestyle, and we hypothesized that mortality would decrease as number of healthy lifestyle factors increased.

**Table 1:** Combined healthy lifestyle scores in the Shanghai Women's Health Study.

Lifestyle Factors Assessed at Baseline	Classification	Scoring Classification	
		Lifestyle Score 1	Lifestyle Score 2
BMI (kg/m^2^)	<18.5, underweight	0	0
	≥30.0, obese	0	0
	25.0–29.99, overweight	1	0
	18.5–24.99, normal weight	2	1
WHR	Tertile 3, ≥0.830	0	0
	Tertile 2, 0.786 to <0.830	1	0
	Tertile 1, <0.786	2	1
Exercise participation (MET h/d)	No activity^a^	0	0
	>0 to 1.99^b^	1	0
	≥2.0^c^	2	1
Spouse smoke	Ever exposed to spouse's smoking	0	0
	Never	1	1
Fruit and vegetable daily intake (g)	Tertile 1, <404.3 g/d	0	0
	Tertile 2, 404.3 to <626.5 g/d	1	0
	Tertile 3, ≥626.5 g/d	2	1

a No exercise participation.

b ∼<30 min of moderate-intensity activity per day.

c ∼≥30 min of moderate-intensity activity per day.

Cox proportional hazards regression models were used to evaluate the associations of mortality with each lifestyle factor individually and then the healthy lifestyle scores. Adjusted hazard ratios (HRs) and their corresponding 95% confidence intervals (CIs) were derived from Cox models after adjusting for potential confounders. Age was used as the time-scale for all models [30], with entry time defined as age at baseline interview and exit time defined as age at death, last follow-up, or December 31, 2007, whichever came first. We first examined associations for each lifestyle factor with mortality adjusted for age and socioeconomic (SES) indicators (occupation [manual and agricultural workers/unknown, clerical, professional], education [≤elementary, junior high school, high school, >high school], and income/person [low, ≤5,000 CNY; middle, 5,000–9999 CNY; high ≥10,000 CNY]). Next, we additionally adjusted for the other lifestyle-factors. Both BMI and WHR remained associated with mortality outcomes after adjustment for each other and the other lifestyle factors; hence, both measures were included in the lifestyle scores. Linear trends were evaluated using the Wald test, treating the lifestyle score as a continuous variable. We examined the proportional hazards assumption, both graphically and by testing the significance of interaction terms for the two lifestyle scores and years of follow-up, and found no evidence for apparent departure from the assumption of proportional hazards.

For healthy lifestyle score 2, we calculated the total population attributable risk (PAR), via summing the exposure-category specific PARs, which estimates the proportion of deaths associated with not having the highest score (i.e., four to five healthy lifestyle-related factors) [31],[32]. We used the following formula to estimate total PARs (percentage), which is appropriate for multicategory exposures and uses adjusted relative risks [31]: 
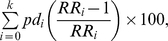
where pd_i_ = proportion of cases in the *i*th exposure level; RR_i_ = relative risks for comparing women with no healthy factors (*i* = 1), 1 healthy factor (*i* = 2), two healthy factors (*i = *3), or 3 healthy factors (*i* = 4), to women with four to five healthy lifestyle factors. PAR estimates are based on the assumption that the observed associations between the lifestyle factors and mortality are causal [31]. All analyses were performed using SAS version 9.2. Tests of statistical significance were based on two-sided probability, and *p*-values<0.05 were considered statistically significant.

## Results

After an average of 9.1 y of follow-up (648,096 person-years), 2,860 deaths were identified among the 71,243 women who reported never smoking or drinking alcohol regularly, including 1,351 from cancer and 775 from CVDs. Compared to women who survived during follow-up, a higher percentage of deceased participants were classified as underweight, overweight or obese, had a higher WHR, reported not participating in exercise regularly, were exposed to spousal smoking, and had a lower daily intake of fruits and vegetables (Table 2).

**Table 2:** Age-adjusted baseline characteristics by survival status in the Shanghai Women's Health Study (*n = *71,243).

**Characteristics**	**Percent Survived (*n = *68,383)**	**Percent Deceased (*n = *2,860)**	***p*-Value^a^**
**Age at baseline (y)**			
40–49	50.7	14.6	
50–59	24.8	19.0	
60–70	24.5	66.4	<0.01
**Education**			
≤Elementary	20.2	26.3	
Junior high school	37.3	41.3	
High school	28.4	22.9	
>High school	14.1	9.5	<0.01
**Occupation**			
Manual and agricultural workers/unknown	50.0	56.6	
Clerical	20.5	21.4	
Professional	29.5	22.0	<0.01
**BMI (kg/m^2^)**			
<18.5	3.3	5.5	
18.5–24.99	61.8	56.2	
25.0–29.99	30.0	31.5	
≥30.0	4.9	6.7	<0.01
**WHR tertiles**			
<0.786	33.5	27.9	
0.786 to <0.830	33.4	30.7	
≥0.830	33.1	41.4	<0.01
**Exercise participation (MET, h/d)**			
None	64.5	66.8	
>0 to <1.99	24.3	23.5	
≥2.0	11.1	9.7	<0.01
**Spouse smoke** b			
Ever	61.0	65.1	
Never	39.0	34.9	<0.01
**Fruit and vegetable intake tertiles (g/d)**			
<404.3	33.1	37.8	
404.3–626.5	33.5	31.2	
≥626.5	33.5	31.0	<0.01

Among women who never smoked cigarettes or drank alcohol regularly. All values (except age) were directly standardized to the age distribution of the cohort.

a *p*-Value from chi-square test for general association.

b Excluded from the analysis were women without information on exposure to spousal smoking (*n = *7,452).

Table 3 shows the HRs for each of the five lifestyle factors with total and cause-specific mortality. In age and SES-adjusted analyses, compared to obese women, those who were normal or overweight had significantly decreased HRs for total mortality, but women who were underweight had a significantly increased HR (Table 3). The association with underweight was no longer significant after excluding deaths in the first 3 y (HR = 1.19; 95% CI 0.93–1.52), suggesting an effect of reverse causation due to weight loss caused by preexisting chronic conditions. HRs for all-cause mortality were significantly decreased for women who had a lower WHR, were physically active, never exposed to spousal smoking, or had higher daily fruit and vegetable intake. Additional adjustment for all the other lifestyle factors did not appreciably change these results, although the associations were attenuated for normal weight, and the HR for spousal smoking status became marginally significant (*p* = 0.061) as shown in Table 3. Similar patterns of associations with WHR were observed for cancer and cardiovascular deaths; findings were less consistent for BMI (Table 3). The patterns of associations with exercise participation, spousal smoking, and fruit and vegetable consumption were comparable to total mortality for deaths from CVD and generally weak or absent for cancer mortality (Table 3).

**Table 3:** Adjusted HRs for lifestyle-related factors and risk of all-cause, cardiovascular, and cancer mortality among nonsmoking and nondrinking women aged 40–70 y at baseline (*n = *71,243), Shanghai Women's Health Study, 1996–2007.

Lifestyle Factor	All-Cause (*n = *2,860 deaths)			CVD (*n = *775)			Cancer (*n = *1,351)		
	*n* Deaths/Cohort	Age and SES-Adjusted^a^ HR (95% CI)	Further Adjusted^b^ HR (95% CI)	*n* Deaths	Age and SES-Adjusted^a^ HR (95% CI)	Further Adjusted^b^ HR (95% CI)	*n* Deaths	Age and SES-Adjusted^a^ HR (95% CI)	Further Adjusted^b^ HR (95% CI)
**BMI (kg/m^2^)**									
≥30 (obese)	257/3,560	1.00 (Reference)	1.00 (Reference)	104	1.00 (Reference)	1.00 (Reference)	99	1.00 (Reference)	1.00 (Reference)
<18.5 (underweight)	149/2,402	1.52 (1.24–1.86)	1.83 (1.48–2.26)	42	1.18 (0.82–1.69)	1.61 (1.10–2.35)	60	1.43 (1.03–1.97)	1.64 (1.18–2.30)
25.0–29.99 (overweight)	1,027/21,328	0.83 (0.72–0.95)	0.85 (0.74–0.98)	299	0.62 (0.50–0.77)	0.64 (0.51–0.80)	483	0.97 (0.78–1.21)	0.99 (0.80–1.23)
18.5–24.99 (normal)	1,427/43,953	0.82 (0.72–0.94)	0.91 (0.80–1.05)	330	0.53 (0.43–0.66)	0.61 (0.48–0.77)	709	0.95 (0.77–1.17)	1.02 (0.82–1.27)
**WHR**									
≥0.830	1,477/23,766	1.00 (Reference)	1.00 (Reference)	447	1.00 (Reference)	1.00 (Reference)	631	1.00 (Reference)	1.00 (Reference)
0.786 to <0.830	811/23,730	0.81 (0.74–0.88)	0.80 (0.73–0.87)	219	0.79 (0.67–0.93)	0.82 (0.69–0.96)	407	0.88 (0.78–1.00)	0.87 (0.76–0.99)
<0.786	572/23,747	0.74 (0.67–0.82)	0.68 (0.61–0.76)	109	0.56 (0.45–0.69)	0.52 (0.42–0.66)	313	0.84 (0.73–0.97)	0.80 (0.69–0.93)
**Exercise participation (MET, h/d)**									
None	1,579/46,093	1.00 (Reference)	1.00 (Reference)	417	1.00 (Reference)	1.00 (Reference)	729	1.00 (Reference)	1.00 (Reference)
>0–1.99	812/17,284	0.91 (0.83–0.99)	0.92 (0.85–1.00)	232	0.90 (0.76–1.06)	0.92 (0.78–1.08)	406	1.04 (0.92–1.18)	1.04 (0.92–1.18)
≥2.0	469/7,866	0.86 (0.77–0.96)	0.89 (0.80–0.99)	126	0.76 (0.62–0.94)	0.79 (0.65–0.97)	216	0.96 (0.82–1.12)	0.96 (0.82–1.12)
**Spouse smoke** c									
Ever	1,315/38,994	1.00 (Reference)	1.00 (Reference)	352	1.00 (Reference)	1.00 (Reference)	634	1.00 (Reference)	1.00 (Reference)
Never	987/24,797	0.92 (0.84–1.00)	0.92 (0.85–1.00)	253	0.84 (0.72–0.99)	0.86 (0.73–1.01)	479	0.93 (0.83–1.05)	0.94 (0.83–1.06)
**Fruit and vegetable intake (g/d)**									
<404.3	1,313/23,742	1.00 (Reference)	1.00 (Reference)	384	1.00 (Reference)	1.00 (Reference)	522	1.00 (Reference)	1.00 (Reference)
404.3 to <626.5	823/23,752	0.83 (0.76–0.91)	0.85 (0.78–0.93)	207	0.78 (0.66–0.92)	0.81 (0.68–0.96)	437	1.03 (0.91–1.18)	1.05 (0.92–1.19)
≥626.5	724/23,749	0.82 (0.75–0.90)	0.85 (0.77–0.93)	184	0.81 (0.68–0.97)	0.84 (0.70–1.00)	392	1.01 (0.89–1.16)	1.03 (0.90–1.18)

a HRs are estimated from Cox proportional hazards regression models using age as the time-scale and adjusted for education, occupation, and income.

b Additionally adjusted for other lifestyle factors in the table.

c Excludes women without information on exposure to spousal smoking (*n = *7,452).

We also considered waist circumference as a measure of central adiposity, however, as compared to WHR, waist circumference was not as strongly associated with mortality outcomes. Compared with the highest waist circumference tertile (≥81 cm), the HRs for total mortality for the lowest tertile (<73 cm) and the middle tertile (73 to <81 cm) were 0.78 (95% CI 0.68–0.89) and 0.89 (95% CI 0.80–0.99), respectively, adjusting for SES indicators, BMI, exercise participation, spouse smoking status, and fruit and vegetable intake. Similar HRs were found for cardiovascular and cancer mortality, although the HRs for the middle tertile of waist circumference were not statistically significant (unpublished data).

A higher healthy lifestyle score 1 was significantly associated with a reduced risk of mortality from all-causes (*p*_trend_<0.01), and from CVD (*p*_trend_<0.01) and cancer (*p*_trend_ = 0.022) (Table 4). For example, women with 7–9 points (most healthy), had a 47% reduction in risk of all-cause mortality (HR = 0.53; 95% CI 0.43–0.63), compared to women with 0–2 points (least healthy). Reductions in mortality associated with a higher lifestyle score were the strongest for deaths due to CVD. Similar patterns were generally seen for healthy lifestyle score 2 and total and cause-specific mortality (Table 4); hence, score 2 was used in subsequent analyses as it is simpler and easier to interpret than score 1. Not having four to five healthy lifestyle factors was associated with total PARs of 33% for total mortality, 59% for CVD mortality, and 19% for cancer mortality (Table 4).

**Table 4:** Healthy lifestyle scores and risk of all-cause, cardiovascular, and cancer mortality among nonsmoking and nondrinking women aged 40–70 y at baseline (*n = *63,791), Shanghai Women's Health Study, 1996–2007.

Lifestyle Score	Percent	All-Cause (*n = *2,302 deaths)			CVD (*n = *605)			Cancer (*n = *1,113)		
		*n* Deaths	HR	(95% CI)	*n* Deaths	HR	(95% CI)	*n* Deaths	HR	(95% CI)
Score 1										
0–2	13.2	507	1.00	(Reference)	163	1.00	(Reference)	204	1.00	(Reference)
3	16.6	465	0.84	(0.74–0.95)	125	0.72	(0.57–0.92)	205	0.89	(0.73–1.08)
4	22.4	497	0.78	(0.69–0.89)	132	0.70	(0.56–0.89)	241	0.88	(0.73–1.06)
5	22.1	415	0.70	(0.62–0.80)	105	0.61	(0.48–0.78)	214	0.82	(0.68–1.00)
6	15.8	270	0.66	(0.57–0.77)	55	0.47	(0.34–0.64)	154	0.85	(0.68–1.05)
7–9	10.1	148	0.53	(0.43–0.63)	25	0.31	(0.20–0.48)	95	0.76	(0.59–0.97)
Trend test				*P*<0.001			*P*<0.001			*P* = 0.022
Score 2										
0	10.8	363	1.00	(Reference)	123	1.00	(Reference)	153	1.00	(Reference)
1	29.9	801	0.93	(0.82–1.05)	219	0.77	(0.62–0.96)	376	1.00	(0.83–1.20)
2	34.6	723	0.84	(0.74–0.95)	180	0.66	(0.52–0.83)	353	0.90	(0.74–1.09)
3	19.5	343	0.75	(0.64–0.87)	72	0.51	(0.38–0.68)	186	0.87	(0.70–1.09)
4–5	5.2	72	0.57	(0.44–0.74)	11	0.29	(0.16–0.54)	45	0.76	(0.54–1.06)
Trend test				*p*<0.001			*p*<0.001			*p* = 0.030
Total PAR^a^				33.4			58.7			18.9

All HRs are estimated from Cox proportional hazards regression models with age as the time-scale and are adjusted for education, occupation, and income. Range for score 1, 0–13 possible points; range for score 2, 0–5 possible points.

a Estimated by summing exposure-category specific PARs from score 0 to 3 using the group with score 4–5 as the reference.

We examined the relation between all-cause mortality and healthy lifestyle score 2 among three subgroups of women classified by their chronic disease history at baseline: (1) women with potentially fatal chronic conditions including cancer, stroke, or coronary heart disease (CHD) (*n = *6,009); (2) women with only less serious conditions, including hypertension and diabetes (*n = *12,209); and (3) healthy women with no history of above-mentioned conditions (*n = *45,573) (Table 5). Results for women in these three groups were fairly similar to overall findings, with significant trends for increasing number of healthy lifestyle factors in each subgroup. We also examined the association of all-cause mortality and healthy lifestyle score 2 by age (<55 y and ≥55 y). Results in these two age groups were similar to overall findings (unpublished data).

**Table 5:** Healthy lifestyle score two and risk of all-cause mortality among nonsmoking and nondrinking women aged 40–70 y by chronic disease status at baseline, Shanghai Women's Health Study, 1996–2007.

Lifestyle Score Two	Women with Potentially Fatal Diseases at Baseline^a^ (*n = *6,009)			Women with Diabetes and Hypertension only at Baseline (*n = *12,209)			Healthy Women at Baseline^b^ (*n = *45,573)		
	*n* Deaths	HR	(95% CI)	*n* Deaths	HR	(95% CI)	*n* Deaths	HR	(95% CI)
0	113	1.00	(Reference)	123	1.00	(Reference)	127	1.00	(Reference)
1	241	1.02	(0.81–1.28)	250	1.02	(0.82–1.27)	310	0.87	(0.71–1.07)
2	186	0.86	(0.67–1.08)	186	0.92	(0.73–1.16)	351	0.87	(0.71–1.07)
3	96	0.82	(0.62–1.09)	69	0.77	(0.57–1.04)	178	0.79	(0.62–0.99)
4–5	17	0.53	(0.32–0.89)	16	0.69	(0.41–1.16)	39	0.61	(0.43–0.88)
Trend test			*p* = 0.005			*p* = 0.024			*p* = 0.009

All HRs are estimated from Cox proportional hazards regression models with age as the time-scale and are adjusted for education, occupation, and income.

a Potentially fatal chronic diseases included cancer, stroke, and coronary heart disease.

b No history of cancer, stroke, coronary heart disease, diabetes, or hypertension.

Sensitivity analyses were conducted to investigate the potential for bias due to the existence of subclinical diseases by excluding deaths occurring in the first 3 y of follow-up. Results from these analyses were similar to those shown in Table 4 for total mortality and mortality due to CVD and cancer. HRs for women with four to five healthy lifestyle factors compared to zero factors were 0.60 (95% CI 0.45–0.80; *p*_trend_<0.01) for total mortality, 0.31 (95% CI 0.16–0.60; *p*_trend_<0.01) for CVD mortality, and 0.75 (95% CI 0.51–1.11; *p*_trend_ = 0.11) for cancer mortality.

**Figure 1:**
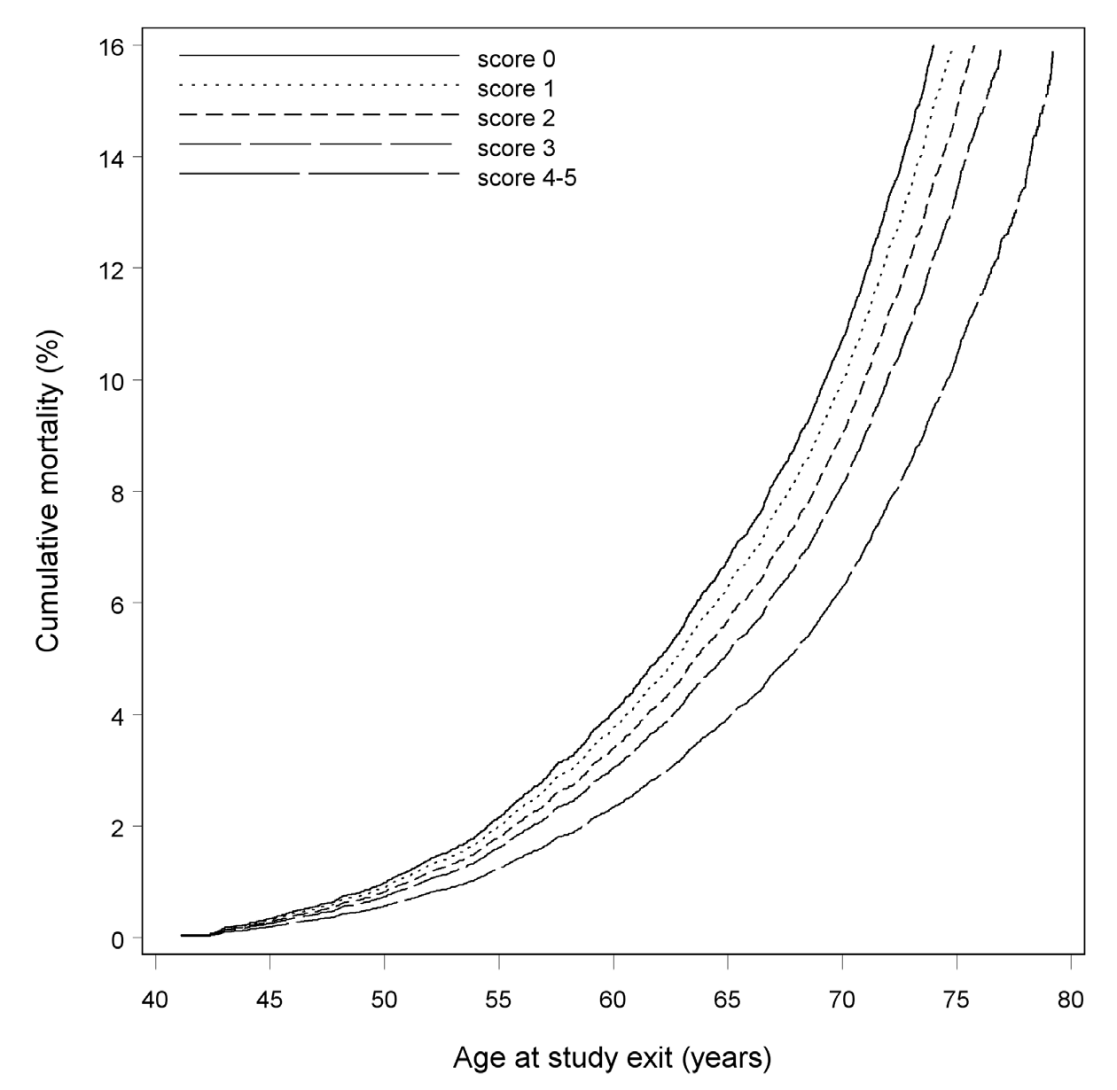
Mortality and healthy lifestyle score 2, adjusted for education, occupation, and income, Shanghai Women's Health Study 1996–2007.

Figure 1 displays cumulative mortality estimates from the Cox proportional hazards regression model with age as the time-scale for score 2, adjusting for education, occupation, and income. The cumulative mortality for the healthy lifestyle score 2 by age at study exit was lowest for women with four to five healthy lifestyle factors and highest for women with zero factors (Figure 1).

## Discussion

In this population-based prospective cohort study of Chinese women aged 40–70 y, we found that healthier lifestyle-related factors—including normal weight, lower WHR, participation in exercise, never being exposed to spousal smoking, and higher daily fruit and vegetable intake—were significantly and independently associated with lower risk of total and cause-specific mortality. Healthy lifestyle scores, composite measures of these five factors, were significantly associated with decreasing mortality as a number of healthy factors increased. The associations persisted for all women regardless of their baseline comorbidities. To our knowledge, this is the first large prospective cohort study specifically designed to quantify the combined impact of lifestyle-related factors on mortality outcomes among lifetime nonsmokers and nonalcohol drinkers. Results show that lifestyle factors other than active smoking and alcohol drinking have a major combined impact on mortality on a scale comparable to the effect of smoking as the leading cause of death in most populations [11],[13],[14].

In general, the literature is limited in regard to the study of combinations of lifestyle factors and mortality [2],[12]–[14],[33]–[38]. Further, most such studies have included alcohol and/or smoking [2],[12],[14],[33]–[37], and little is known about the combined impact of lifestyle factors other than active smoking and drinking in relation to mortality. The answer to this question is of particular importance as there are a substantial number of people worldwide who are nonsmokers and do not drink excessively [14],[39]. In an attempt to address this question, in a subgroup analysis among never-smokers in the Nurse's Health Study, van Dam and colleagues reported a 2-fold excess risk of all-cause mortality among women who had a high BMI, low physical activity, and unhealthy diet [13]. That study, however, did not consider environmental tobacco smoke or measures of central adiposity such as WHR.

Another limitation of previously published studies is that most studies have been conducted in the United States or Western Europe, and few studies have examined the combined impact of lifestyle factors in relation to mortality among Asian populations. We did, however, identify three reports from Japan, two conducted in rural northern Japan [34],[37] and one among individuals of the Japan Collaborative Cohort Study [12]. Each of these reports demonstrated that healthier lifestyles based on several lifestyle-related factors were associated with substantial reductions in death among Asian men and women. None of these reports, however, focused on evaluating the impact of lifestyles on mortality outcomes among nonsmokers and nondrinkers.

To our knowledge, this is the first investigation of combinations of lifestyle factors and risk of mortality among Chinese women. We selected five factors that are easy to assess and interpret, based both on prior knowledge of lifestyle factors in relation to mortality and public health recommendations [1],[4]–[9],[18]–[24]. BMI, exercise participation, and fruit and vegetable intake have been well-studied in relation to mortality [1],[5]–[9],[24]. WHR and environmental tobacco smoke have not been studied as much, but evidence is accumulating for these two factors as important predictors of total mortality [3],[4],[23],[40],, and both were shown to be associated with mortality among SWHS participants [18],[21]. Several large prospective cohort studies among women have shown WHR to be an important predictor of mortality independent of BMI [3],[4],[18],[41], and in some populations, WHR may be an even stronger predictor of mortality [4],[41]. Hence, on the basis of previous studies that both BMI and WHR may be independent measures of adiposity among women and our findings for independent effects of BMI and WHR after adjustment for each other and additional potential confounders, we included both BMI and WHR in the lifestyle scores. In addition, environmental tobacco smoke is a particularly important exposure for women living in China and other Asian countries given the high smoking prevalence among Asian men [21],[42]. No previous study included either WHR or environmental tobacco smoke in the assessment of the combined impact of lifestyle factors on mortality.

This study has several strengths, including a population-based prospective cohort study design and large overall sample size. Selection bias was minimized due to the exceptionally high response rates at recruitment (92.7%) and in the follow-up surveys (96.7%–99.8%). Baseline assessments were conducted by trained interviewers using standardized protocols, and anthropometric data were based on measurements instead of self-report.

Limitations of this study should be considered for interpretation of results. One concern is the potential for information or reverse causation bias due to the presence of subclinical disease or prevalent clinical disease. To address this concern, we analyzed the association of mortality with the lifestyle score among women without prevalent CVD, cancer, stroke, diabetes, or hypertension and also after excluding deaths in the first 3 y of follow-up. Findings for these subgroups were not appreciably different from the overall results. Women without information on exposure to spousal smoking were excluded from the lifestyle score and mortality analyses. Exclusion of these women, however, is unlikely to materially affect our findings, though the sample size was reduced slightly. Measurement error, particularly for self-reported data on diet and exercise, is another potential concern. However, we have previously shown good validity and reliability for diet and physical activity data from the SWHS [25],[26]. Furthermore, nondifferential errors tend to attenuate the observed associations, and thus the true association between lifestyle factors and mortality may be stronger than that estimated in this study. We did not adjust for potential mediators such as blood lipid levels and hypertension in the analysis since the primary purpose of the study was to quantify the overall impact of lifestyle on mortality outcomes. Adjustment for mediators in the causal pathway between lifestyle factors and mortality would affect the quantification of the overall impact of these lifestyle factors on mortality outcomes.

For ease of interpretation, healthy lifestyle scores were created in the analysis assuming an equal weight for each of the factors included. A weighted approach based on the effect size of each variable could improve the estimate of the overall impact of lifestyle factors on mortality. However, as demonstrated in our study, the estimates using score 1 (semi-weighted) and score 2 (nonweighted) are similar, suggesting that a weighted approach may not improve the estimates substantially. Despite an overall large sample size, the sample sizes for some cause-specific analyses were relatively small, which may affect the precision of the point estimates. In addition, the observed associations between lifestyle factors and mortality outcomes in our study may be underestimated because of the use of baseline covariate measurements only [43]. Extended follow-up of this cohort will provide the opportunity to further evaluate the impact of these lifestyle-related factors on mortality outcomes in the future.

Most of the lifestyle-related factors studied here may be improved by individual motivation to change unhealthy behaviors. For example, changes in physical activity levels and energy expenditure to reduce adiposity can be made by increasing activity levels through walking daily or participating in group exercise classes. Increased fruit and vegetable intake is fairly straightforward for the majority of Chinese women in urban communities, given that many varieties of fruit and vegetables are readily available at the markets. However, both the physical and social environments also are important contributors to sustained lifestyle changes, and may be more significant than individual motivation for some lifestyle factors, which is particularly true for exposure to spousal smoking. Change in exposure to spousal smoking may start with increased awareness by both women and their husbands about the detrimental health effects of smoking, but also will require community-based interventions and change in the social environment (e.g., promotion of home smoking bans in communities) [44].

In conclusion, in this first study to quantify the combined impact of lifestyle-related factors on mortality outcomes among Chinese women, we found that a higher healthy lifestyle score based on five factors was associated with substantial reductions in total and cause-specific mortality among lifetime nonsmoking and nondrinking women. Reductions in premature deaths associated with higher healthy lifestyle scores were seen among women with and without preexisting comorbidities. Our study suggests that a combined healthy lifestyle—including being of normal weight, lower central adiposity, participation in physical activity, nonexposure to spousal smoking, and higher fruit and vegetable intake—can result in lower mortality among middle-aged and older women, including women with a history of severe disease. Research is needed to design appropriate interventions to increase these healthy lifestyle factors among Asian women.

## Supporting Information

Table S1 Baseline characteristics for study participants before and after exclusions, Shanghai Women's Health Study.
(0.08 MB DOC)

## Abbreviations

BMI: body mass index
CI: confidence interval
CVD: cardiovascular disease
HR: hazard ratio
MET: metabolic equivalent
PAR: population attributable risk
SES: socioeconomic
SWHS: Shanghai Women's Health Study
WHR: waist-hip ratio
