# Supplementary material for: Combined Impact of Lifestyle-Related Factors on Total and Cause-Specific Mortality among Chinese Women: Prospective Cohort Study
Source: PLoS Med. 2010 Sep 14;7(9):e1000339. doi: 10.1371/journal.pmed.1000339 (PMC2939020; doi:10.1371/journal.pmed.1000339)
Supplement: Table S1 — Baseline characteristics for study participants before and after exclusions, Shanghai Women's Health Study. (0.08 MB DOC) [file pmed.1000339.s001.doc]

| **Table S1.** Baseline characteristics for study participants before and after exclusions, Shanghai Women’s  Health Study | | | | | | | |
| --- | --- | --- | --- | --- | --- | --- | --- |
| Characteristics | All Participants  (n=74,942) |  | Exclusions  (1)^*^  (n=71,243) | |  | Exclusions  (2)^‡^  (n=63,791) | |
|  | (%) |  | (%) | P-value^†^ |  | (%) | P-value^†^ |
| Age at baseline (years) |  |  |  |  |  |  |  |
| 40-49 | 48.5 |  | 49.2 |  |  | 51.8 |  |
| 50-59 | 24.3 |  | 24.6 |  |  | 25.2 |  |
| 60-70 | 27.2 |  | 26.2 | <0.001 |  | 23.1 | <0.001 |
| Education |  |  |  |  |  |  |  |
| ≤Elementary | 21.6 |  | 21.1 |  |  | 20.3 |  |
| Junior high school | 36.9 |  | 36.9 |  |  | 37.2 |  |
| High school | 27.9 |  | 28.1 |  |  | 28.4 |  |
| >High school | 13.6 |  | 13.9 | 0.003 |  | 14.0 | <0.001 |
| Occupation |  |  |  |  |  |  |  |
| Manual and agricultural  workers/unknown | 50.8 |  | 50.4 |  |  | 50.1 |  |
| Clerical | 20.7 |  | 20.5 |  |  | 20.3 |  |
| Professional | 28.5 |  | 29.1 | 0.002 |  | 29.6 | <.0001 |
| Body mass index (kg/m^2^) |  |  |  |  |  |  |  |
| <18.5 | 3.4 |  | 3.4 |  |  | 3.3 |  |
| 18.5- 24.99 | 61.4 |  | 61.5 |  |  | 61.4 |  |
| 25.0-29.99 | 30.0 |  | 30.1 |  |  | 30.2 |  |
| ≥30.0 | 5.1 |  | 5.1 | 0.99 |  | 5.0 | <.0001 |
| Waist-hip ratio tertiles |  |  |  |  |  |  |  |
| <0.786 | 33.0 |  | 33.1 |  |  | 33.0 |  |
| 0.786-<0.830 | 33.2 |  | 33.3 |  |  | 33.3 |  |
| ≥0.830 | 33.8 |  | 33.6 | 0.53 |  | 33.7 | 0.83 |
| Exercise participation    (MET-h/d) |  |  |  |  |  |  |  |
| None | 64.5 |  | 64.1 |  |  | 64.5 |  |
| >0-<1.99 | 24.3 |  | 24.4 |  |  | 24.4 |  |
| ≥2.0 | 11.3 |  | 11.2 | 0.44 |  | 11.1 | 0.28 |
| Fruit and vegetable intake tertiles (g/day) |  |  |  |  |  |  |  |
| <404.3 | 33.8 |  | 33.5 |  |  | 33.2 |  |
| 404.3-626.5 | 33.1 |  | 33.3 |  |  | 33.7 |  |
| ≥626.5 | 33.2 |  | 33.2 | 0.25 |  | 33.1 | 0.0009 |
